# Doppler Echocardiography Assessment of Coronary Microvascular Function in Patients With Angina and No Obstructive Coronary Artery Disease

**DOI:** 10.3389/fcvm.2021.723542

**Published:** 2021-10-29

**Authors:** Jakob Schroder, Eva Prescott

**Affiliations:** Department of Cardiology, Bispebjerg Frederiksberg Hospital, University of Copenhagen, Copenhagen, Denmark

**Keywords:** coronary flow velocity reserve, stress echocardiography, coronary microvascular dysfunction, prognosis, sex

## Abstract

Echocardiographic evaluation is an essential part of the diagnostic work-up in patients with known or suspected cardiovascular disease. Transthoracic Doppler echocardiography (TTDE) enables straightforward and reliable visualization of flow in the left anterior descending artery. In the absence of obstructive coronary artery disease, low TTDE-derived coronary flow velocity reserve (CFVR) is considered a marker of coronary microvascular dysfunction (CMD). TTDE CFVR is free from ionizing radiation and widely available, utilizing high-frequency transducers, pharmacologic vasodilator stress, and pulsed-wave Doppler quantification of diastolic peak flow velocities. European Society of Cardiology guidelines recommend TTDE CFVR evaluation only following preceding anatomic invasive or non-invasive coronary imaging excluding obstructive CAD. Accordingly, clinical use of TTDE CFVR is limited and CMD frequently goes undiagnosed. An evolving body of evidence underlines that low CFVR is an important and robust predictor of adverse prognosis and continuing symptoms in angina patients both with and without obstructive CAD. The majority of angina patients have no obstructive CAD, particularly among women. This has led to the suggestion that there may be a gender-specific female atherosclerotic phenotype with less epicardial obstruction, and a low CFVR signifying CMD instead. Nevertheless, available evidence indicates low CFVR is an equally important prognostic marker in both men and women. In this review, TTDE CFVR was evaluated regarding indication, practical and technical aspects, and interpretation of results. Association with symptoms and prognosis, comparison with alternative invasive and non-invasive imaging modalities, and possible interventions in angina patients with low CFVR were discussed, and key research questions were proposed.

## Introduction

Coronary artery disease remains one of the leading causes of morbidity and mortality in Western countries in both men and women, with male sex as a risk factor for the early development of coronary artery disease (CAD) (1, 2). The present diagnostic paradigm in patients with angina pectoris is focused on likelihood and subsequent identification of obstructive CAD (3, 4), but most patients referred for assessment do not fulfill criteria for invasive coronary angiography (ICA), and in the subset of patients ultimately examined with ICA, many patients, especially women, have no obstructive CAD. Ultimately, only a fraction of angina patients is treated with revascularization (5–7). In effect, a treatable etiological explanation remains absent in many patients with angina, particularly in women.

More than 30 years ago, efforts to measure coronary flow velocities in the coronary epicardial arteries with Doppler echocardiography began (8, 9). Early proof of concept studies established a high degree of correlation between invasively derived flow measurements and values obtained *via* transesophageal, and later transthoracic Doppler echocardiography (TTDE) (10–12). TTDE was considered a promising imaging modality given that echocardiography was already a principal part of the diagnostic work-up in nearly all patients suspected of cardiac disease. Initial studies aimed at determining the potential presence and degree of coronary epicardial stenosis (13–15). Later on, the focus shifted toward visualization and quantification of the most accessible coronary branch, i.e., the left anterior descending (LAD) artery, during rest and pharmacologic stress as an indirect measure of the perfusion capacity of the entire coronary circulation (13, 16, 17). Vasodilation in the microvascular compartment is the main determinant of increased coronary blood flow during exertion. In the absence of any significant epicardial stenosis, changes in coronary flow velocity relative to resting flow is considered an indirect measure of the coronary microvascular function, i.e., coronary flow velocity reserve (CFVR) (17, 18).

The relevant body of evidence related to TTDE CFVR in angina patients with no obstructive CAD consists of a combination of (i) studies in selected patient cohorts with no obstructive CAD and (ii) studies performed in mixed patient populations both with and without obstructive CAD. We had chosen to include both study types, while clearly highlighting possible interpretation difficulties and limitations of the evidence related to the mixed nature of the patient populations in the latter group.

In this review, we evaluated TTDE CFVR regarding indication, practical and technical aspects, and interpretation of results in patients with angina and no obstructive coronary artery disease (ANOCA). Moreover, association with symptoms and prognosis, comparison with alternative invasive and non-invasive imaging modalities, and possible interventions in angina patients with low CFVR were discussed. Finally, we proposed a possible translation of TTDE CFVR to the clinic and questions in relation to angina patients and CFVR that remain unanswered.

## Pathophysiologic Basis

Under resting conditions, the coronary blood flow is kept constant at varying coronary pressures. During exertion, coronary blood flow is increased due to a simultaneous increase in coronary perfusion pressure and a decrease in coronary vascular resistance. Vasodilation is primarily induced at the level of small arteries and precapillary arterioles *via* several regulatory mechanisms acting in conjunction and interdependent mutual stimulation (19–21). Vasodilatory mechanisms include the flow-mediated endothelium-dependent release of nitric oxide, a myogenic response to increased transmural arteriolar pressure, metabolic regulation triggered by local alterations in the levels of vasoactive metabolites, e.g., carbon dioxide and adenosine inducing non-endothelium dependent vasodilation, and neural sympathetic stimulation of β-receptors also contributes to decreased vascular resistance under conditions of increased oxygen demand (17, 22–24). In healthy subjects coronary blood flow may be increased 4- to 6-fold during maximal exertion. This capacity for coronary vasodilation may be expressed as the ratio between peak and baseline perfusion, the coronary flow reserve (CFR) (9, 14, 19). In patients with unobstructed epicardial coronary arteries, these mainly serve the purpose of conduit vessels transporting blood to the smaller vessels, which regulate the vascular resistance as described above. Conversely, in patients with epicardial obstructive CAD, the CFR will be reduced in accordance with the degree of stenosis (17, 19).

It is not possible to visualize the microcirculation *in vivo*, but in patients with no significant epicardial flow-limiting stenosis (<50% stenosis and fractional flow reserve >0.8 at ICA) a reduced CFR is considered a marker of reduced vasodilatory capacity in the small coronary arteries and arterioles, i.e., coronary microvascular dysfunction (CMD) (16, 17, 19, 25). Pathophysiologically, CMD may be due to both structural and functional alterations including capillary rarefaction, decreased arteriolar lumen/wall ratio, and impaired endothelium- and non-endothelium dependent vasodilatation or excessive adrenergic vasoconstriction, often with several abnormal findings present in the same patient. Furthermore, CMD may be present both in patients with and without obstructive CAD or myocardial disease (21, 26–29).

Different vasodilatory and adrenergic agents induce a near-maximal increase in coronary blood flow during CFR quantification, partly due to indirect concomitant activation of both hemodynamic, metabolic, and neural mechanisms. Direct quantification of coronary flow reserve of the entire myocardium can be measured by positron emission tomography (PET). Quantification of flow reserve in any epicardial vessel is obtainable during ICA. TTDE visualization of the main coronary arteries allows assessment of coronary flow velocity as an alternative to absolute flow (16, 25, 29). Under the assumption that the epicardial vessel lumen is kept relatively constant during pharmacologic stress, the change in coronary flow velocity from baseline to maximal hyperemia is considered directly proportional to the change in absolute flow, thereby allowing the calculation of a CFVR during vasodilator-stress TTDE as a proxy for CFR (8, 9, 30, 31). TTDE CFVR assessment of coronary microvascular function is based on quantification of the relative increase in coronary perfusion during pharmacologic stimulation of non-endothelium dependent vasodilation pathways and indirect concomitant activation of other hemodynamic and neural mechanisms (21, 26). Evaluation of endothelium-dependent vasodilation capacity and macro- or micro-vascular vasospasm, which both require intracoronary acetylcholine infusion, are not assessed during TTDE CFVR.

## TTDE CFVR Examination Methodology

**Figure 1:**
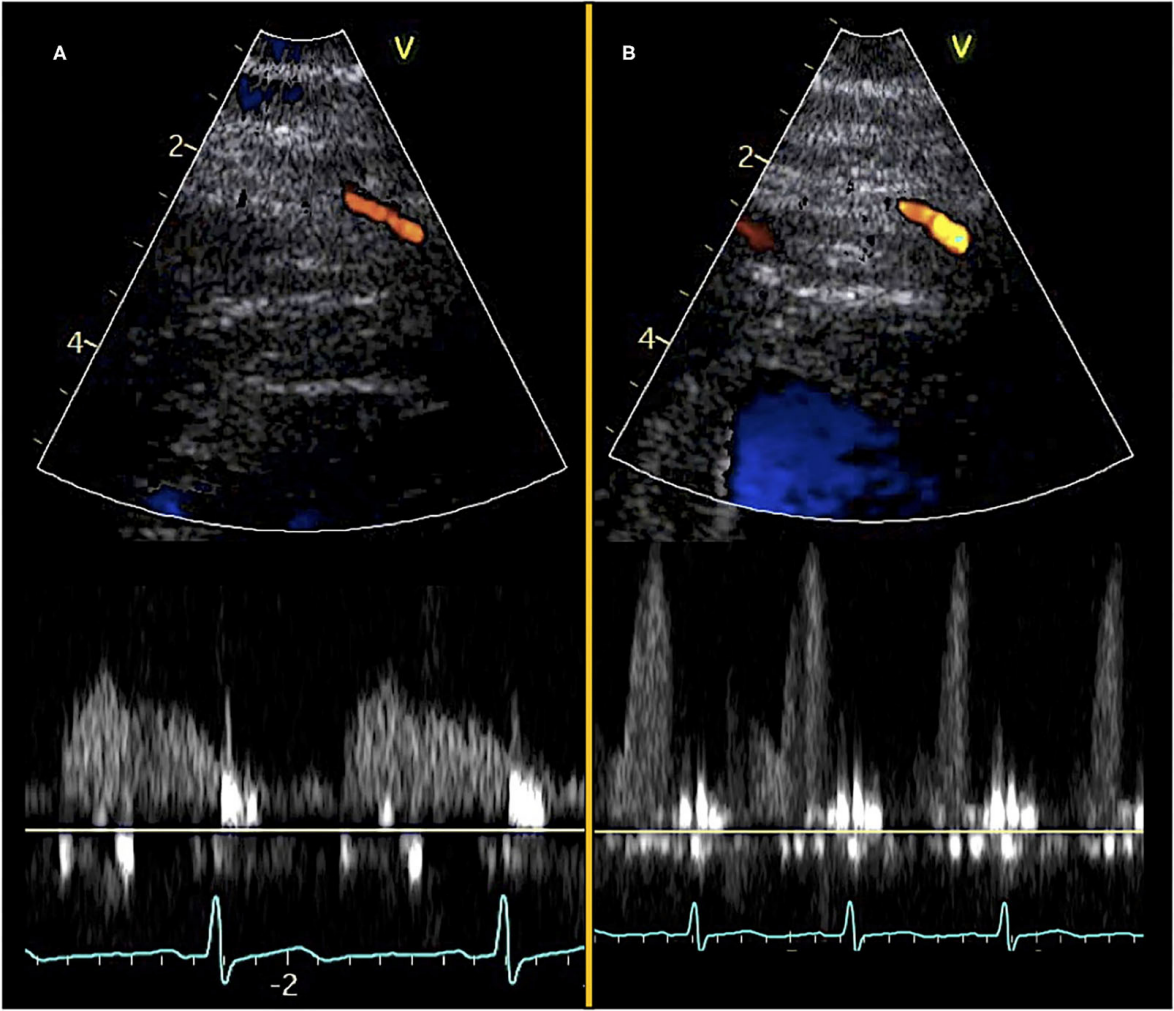
Transthoracic Doppler echocardiography and pulsed-wave Doppler curves. Color Doppler visualization of mid-distal LAD (top) and diastolic pulsed-wave flow velocity curves (bottom). **(A)** Images obtained at rest and **(B)** images obtained during adenosine stress.

Coronary flow velocity measurements may be obtained in several of the larger coronary arteries. However, in the context of CMD evaluation, the assessment is most commonly performed *via* identification of the mid-distal LAD due to its position near the chest wall, thus providing reliable and optimal images and flow curves. Nonetheless, if LAD visualization is difficult in selected patients, measurements may also be obtained in other larger coronary branches (8, 16, 25, 30, 32, 33). The TTDE examination may be performed with commercially available ultrasound machines using a phased array high-frequency ultrasound probe usually in the range from >3 to 8 MHz with harmonic imaging to obtain high-resolution color Doppler visualization of the mid-distal LAD (8, 9, 12, 30, 34). The patient is studied in the left lateral decubitus position. A baseline color scale of ~1–2.5 kHz (velocity range of ± 10–24 cm/s) may be used as a standard for obtaining the color Doppler. The mid-distal LAD is usually located in the interventricular sulcus at the midway between a foreshortened two- and three-chamber apical view. However, due to anatomic variations, the use of modified and apical views is often necessary to obtain optimal LAD visualization. Diastolic maximal coronary flow velocities are measured by pulsed-wave Doppler as a flow signal in the LAD toward the transducer (Figure 1, Supplementary Videos 1, 2). The coronary Doppler flow profile is biphasic with diastolic predominance. To avoid systolic motion artifacts and to achieve reproducible maximal velocities the diastolic flow is used for CFVR calculation. The blood flow direction of the LAD is adjusted to be close to parallel with the direction of the pulsed-wave Doppler ultrasound beam and a 3–4 mm sample volume is positioned over the LAD color flow. Sample volume size is adjusted as needed to balance signal intensity and noise. In case of unsatisfactory quality of LAD color signal or flow velocity profile, it is also possible instead to visualize and assess flow in either the right coronary artery (RCA) or posterior descending artery in the posterior interventricular groove (modified two-chamber view) or the circumflex coronary artery (Cx) in the basal part of the lateral left ventricular wall (apical four-chamber view) (33, 35–38). The deeper position of these branches may necessitate the use of an echo probe with a slightly lower frequency range <6 Mhz. Furthermore, if it is not possible to locate the LAD or other coronary arteries due to low image quality or anatomic variation a Doppler echo contrast agent may be infused to increase image quality and Doppler signal, taking care not to misinterpret exaggerated enhancement of the LAD Doppler signal. Current commercially available echo contrast agents used at some centers include sulfur hexafluoride and perflutren, among others (39–43).

Care must be taken to ensure coronary flow velocity measurements during rest and vasodilator stress are performed on the same segment of the coronary artery with minimal angle error. Additionally, any unavoidable remaining angle error should be similar at rest and stress thereby making the angle error theoretically redundant. The pulsed-wave Doppler tracing should have a clear outer edge. Resting coronary flow velocity should be the mean of three consecutive cardiac cycles (>6 if atrial fibrillation or other irregular heart rhythms). Stress coronary flow velocity should be the highest value obtained during vasodilator infusion and three consecutive cardiac cycles should preferably be obtained, although this is sometimes challenging due to cardiac motion and hyperventilation during stress. The patient may be instructed to hold their breath to limit hyperventilation artifacts (44). Readings of coronary flow velocity values have been shown to have good inter- and intra-observer variability and low coefficient of variation in the range of 5–10% (8, 11, 43, 45–49).

The pharmacologic stress agents used in CFVR evaluation are also used to achieve maximal perfusion in other stress imaging modalities, e.g., PET, cardiac MRI (cMRI) perfusion, and single-photon emission computed tomography (16, 50). The first choice agents in CFVR examination are the vasodilators adenosine and dipyridamole (16, 51–54). Adenosine acts by directly stimulating the A_2A_-receptors on vascular smooth muscles cells in the microvascular vessel wall eliciting relaxation and vasodilation. Dipyridamole stimulates the same pathway indirectly by inhibiting adenosine deaminase and phosphodiesterase, effectively increasing the adenosine concentration in the vessel wall (8, 9, 19, 21). Importantly, these non-endothelium-dependent mechanisms do not cause any direct vasodilation in the larger epicardial arteries (55, 56). It has been shown that administration of intracoronary or sublingual nitroglycerine prior to stress testing dilates the epicardial vessels resulting in a reduced resting coronary flow velocity without an increase in stress coronary flow velocity, ultimately yielding a higher CFVR ratio (57, 58). However, pre-stress nitroglycerine has not been used in any larger clinical studies employing adenosine/dipyridamole stress imaging.

The recommended dose of adenosine stress is 0.14 mg/kg/min, and high-dose dipyridamole stress (0.84 mg/kg over 6 min) produces comparable values of CFVR although maximal stress coronary flow velocity values are possibly only achieved with adenosine (8, 52). Due to the rapid onset of action of adenosine, maximal flow velocity may be recorded quickly after the infusion has started, usually after 1–2 min, while maximal coronary flow velocity using dipyridamole is usually obtained after 3–6 min. Most CFVR protocols perform continuous coronary flow velocity stress measurements during ~5–7 min of vasodilator stress. The main contraindication for adenosine/dipyridamole is the presence of severe hypotension, a reactive pulmonary obstructive disease that may be aggravated during adenosine infusion and inadvertent stimulation of A_2B_ adenosine receptors in small airways, and cardiac conduction abnormalities, such as higher degree atrioventricular block, which may be aggravated *via* stimulation of A_1_ adenosine receptors (50, 52, 59, 60). Any side effects of adenosine rapidly diminish due to its short half-life, whereas the longer half-life for dipyridamole often necessitates intravenous administration of refractory doses of an antagonizing methylxanthine, e.g., theophylline and aminophylline (30, 61).

The adrenergic agonist dobutamine is often used for SE in the evaluation of wall motion abnormalities due to its positive inotropic and chronotropic effects. However, in addition to increasing coronary flow *via* adrenergic stimulation, it also dilates the epicardial coronary arteries *via* β-adrenergic activation, resulting in the more difficult interpretation of coronary flow velocity changes. The increase in contractility makes consistent measures of coronary flow more difficult. Accordingly, dobutamine stress in CFVR evaluation is mainly suggested as an alternative if adenosine/dipyridamole are contraindicated due to comorbidity (56, 62, 63).

Regadenoson is a novel A_2A_-selective adenosine receptor agonist which causes vasodilation with a lower risk of bronchospasm or atrioventricular conduction delays as A_1_ and A_2B_ adenosine receptors are not stimulated. Regadenoson is administered as a weight-unadjusted fixed-dose bolus rapidly inducing maximal vasodilation which lasts for ~2–3 min (64, 65). Regadenoson has been suggested as a potential new agent in vasodilator stress testing, but it has not yet been used in larger clinical studies evaluating TTDE CFVR (66–68). Current limitations that it faces include its price and a lack of overview of all possible adverse effects, and up to now most clinical research has been done with non-echocardiography modalities (69, 70).

Abstinence from specific foods, drinks, and medications prior to examination is a prerequisite to gain reliable CFVR measurements. Methylxanthines are competitive inhibitors of adenosine receptors and may significantly attenuate the coronary vasodilator effect of all stress agents acting *via* A_2A_-receptors, such as adenosine, dipyridamole, and regadenoson. Therefore, drinks and foods containing significant amounts of methylxanthine, e.g., coffee, tea, cola, chocolate, and banana, as well as medication used in the treatment of obstructive pulmonary disease containing theophylline must be withheld 12–24 h prior to examination. Any medication containing dipyridamole must be withheld >48 h prior to testing to avoid both an increased risk of side effects during vasodilator stress and an increase in resting flow velocities which would result in an attenuated CFVR ratio (30, 71–73). Nitrates have been shown to cause coronary vasodilation resulting in decreased resting coronary flow velocity, but the exact effect of nitrates on vasodilator stress is not clear. In the context of CFVR evaluation long- and short-acting nitrates should be withheld >24 and >1 h, respectively, prior to vasodilator stress (30, 57, 58). Regarding beta-blockers, dihydropyridine calcium channel blockers, and angiotensin-converting enzyme inhibitors/angiotensin II inhibitors, clinical studies examining the effect on CFVR during vasodilator stress had been small-scale and to some degree contradictory. In general, it has been proposed that these agents may increase CFVR values, thereby lowering the test sensitivity for the detection of reduced CFVR. However, some studies found no effect or a decrease in CFVR during treatment (73–78). We recommend withholding anti-ischemic agents and antihypertensive medication 24 h prior to vasodilator stress unless this causes unacceptable symptoms in the patient, with the aim of reducing uncertainty in the interpretation of CFVR values.

## Current Indication

Contemporary European and North American cardiologic society guidelines suggest clinical utilization of TTDE CFVR only in a limited subgroup of angina patients. The 2019 European Society of Cardiology (ESC) Guideline on Chronic Coronary Syndromes recommended non-invasive assessment of microcirculatory function with TTDE CFVR as a recommendation IIb and evidence level B. Furthermore, it is only recommended in patients with both (i) clear-cut angina, (ii) an abnormal non-invasive functional test [stress echocardiography (SE), single-photon emission computed tomography, PET, or stress cMRI], and (iii) epicardial coronary vessels that are normal or have only mild stenosis at coronary computed tomography angiography or ICA (3). These criteria are in line with the consensus statement from the Coronary Vasomotion Disorders International Study Group (COVADIS) for definitive microvascular angina (MVA) which also necessitates objective demonstration of myocardial ischemia on standard non-invasive functional testing (79). Accordingly, implementation of TTDE CFVR in the large group of angina patients with a normal non-invasive functional test is not supported in the ESC Guideline at present. Furthermore, the Guideline does not mention any sex-specific differences pertaining to the use of TTDE CFVR, although it is mentioned that the development of CMD often precedes the development of epicardial lesions, especially in women (3). The American College of Cardiology Guideline on Stable Ischemic Heart Disease briefly mentions the possibility of perfusion imaging during SE, but in essence, it is underlined that the use of TTDE CFVR in the United States is limited, and there are no specific recommendations regarding TTDE CFVR application (80, 81). Likewise, a recent review of imaging techniques to assess microvascular dysfunction emphasizes PET and possibly cMRI as the most promising candidates for clinical CMD evaluation in the US due to more data and local expertise compared with TTDE CFVR (82).

In contrast to the brief coverage of TTDE CFVR in guidelines, an increasing body of White Papers, reviews, and editorials are proposing a reappraisal of the clinical utilization of coronary microvascular assessment (16, 25, 83–86). A role is proposed in angina patients in whom obstructive CAD has been ruled out by anatomical imaging, regardless of results from functional tests, as a part of the diagnostic work-up in angina patients who are never evaluated with ICA (16, 25, 83–86). Furthermore, several large studies performed at centers already utilizing SE for wall motion scores index as a non-invasive test for risk stratification indicate CFVR evaluation adds incremental prognostic value, both in patients with previous or current obstructive CAD and with no history of obstructive CAD and a normal non-invasive test (67, 68, 87, 88). These studies implying a possibly important role for evaluation of microvascular function have not yet resulted in notable guideline recommendations.

## Angina Pectoris Symptoms

Symptom characteristics, together with age, gender, and risk factors, play a prominent role when determining the clinical likelihood of obstructive CAD in stable angina patients. The rationale for this approach is a relatively strong association between typical angina symptoms and obstructive CAD (3). The symptom characteristics of MVA are generally not thought to differ from those observed in patients with obstructive CAD, i.e., symptoms are most often provoked by exertion, cold or emotional stress, but may also occur at rest and appear in the form of angina equivalents, e.g., shortness of breath (25, 79, 89). However, few studies have directly compared symptoms in angina patients with and without CMD (90, 91). In 1684 women with angina and no obstructive CAD at ICA, a low CFVR indicating CMD was not associated with typical angina characteristics nor severity. It was additionally found that a positive non-invasive diagnostic test for regional ischemia did not predict a low CFVR. The lack of concordance between abnormal functional testing and CMD has also been reported elsewhere (90). These findings challenge the notion that CMD is necessarily a condition distinguished by typical angina symptoms and a positive non-invasive diagnostic test. Indeed, functional testing is aimed at diagnosing regional ischemia caused by obstruction of an epicardial vessel whereas CMD causes patchy ischemia that does not necessarily lead to abnormal functional imaging. A recent study employing 24-h ambulatory ECG monitoring in women with CMD found that asymptomatic electrocardiographic ischemic episodes were frequent, while on the other hand, most patients reported symptoms that were not accompanied by ischemic ECG changes, further indicating the relation between angina severity, characteristics, CMD presence and objective signs of ischemia may be more complex than previously assumed (91, 92).

Regarding acute angina symptoms, the recently updated ESC Guidelines for the management of acute coronary syndromes in patients presenting without persistent ST-segment elevation emphasize ICA remains the reference standard for any high-risk patient regardless of sex (4). However, after rule-out of life-threatening causes of chest pain, the majority of these intermediate-risk patients are observed in Emergency Observation Units and are often discharged with a diagnosis of non-specific or unexplained chest pain (16, 93). Recent studies suggest that up to 40% of patients with acute chest pain have functional signs of CMD. It has been suggested that non-invasive evaluation of possible CMD may be applicable and cost-effective in this group, especially in the context of prior negative non-invasive diagnostic tests, no non-cardiac cause of chest pain, and typical angina symptoms (94, 95). So far, there have not been any TTDE-based studies evaluating the prevalence of low CFVR in Emergency Department patients. Given that standard transthoracic echocardiography is already indicated in most of these patients to rule out structural cardiac disease, the potential add-on of CFVR evaluation may be considered to attain an early definitive diagnosis in a significant proportion of these patients, enabling undelayed initiation of both symptom management and attention to risk factors. Naturally, these potential benefits must be weighed against the risk of burdening the staff of the Emergency Departments, and proof of concept has not been demonstrated in clinical studies.

## Prognosis

Observational studies have shown a greater risk of major adverse cardiovascular events (MACE) in patients with ANOCA compared with asymptomatic peers, in some studies even similar to those with obstructive CAD (6, 96). These results have given rise to several larger studies in the last decade investigating whether the presence of low CFVR is an important risk factor in addition to established risk factors such as hypertension, diabetes mellitus and dyslipidemia (Table 1) (66–68, 87, 88, 97). We report on prognostic studies performed both in patients with no obstructive CAD and mixed population studies also including patients with obstructive CAD. In our description and analysis of the latter, we emphasized limitations pertaining to the combination of patient subtypes and attempt to extract the specific results for the subgroup of patients with no obstructive CAD.

**Table 1:** Prognostic studies in angina patients using TTDE CFVR.

**Author**	**Year**	***N*** **, all**	***N*** **, non-obstructive**	**Men**	**Patients**	**CFVR cut-off**	**Follow-up (median)**	**Primary outcome**	***N*** **, Events**	**Stress agent**	**Effect size**	**Effect women/men**	**Comment**
Nakanishi	2012	272	170	67%	Suspected and known CAD	<2.4	4.0 years	CVD death, MI, UAP, HF	32	Adenosine	5.56 (OR)	Not directly reported^a^	100 had significant Cx or RCA stenosis
Lowenstein	2014	651	651	51%	Suspected and known CAD	<2.0	2.9 years	CVD death, MI, Revascularization	48	Dipyridamole Dobutamine	4.20 (HR)	Not directly reported^a^	RWMA used to assess underlying stenosis
Cortigiani	2019	5,577	3,524 without prior CAD^b^	59%	Suspected and known CAD	≤ 2.0	1.7 years	All-cause death, MI	649	Dipyridamole	3.26 (HR)	Not directly reported^a^	Specific assessment of optimal cut-off in subgroups
Ciampi	2019	1,867	n/a	n/a	Suspected and known CAD	≤ 2.0	1.3 years	All-cause death, MI, stroke, revascularization, HF admission	218	Exercise Adenosine Dipyridamole Dobutamine	1.60 (HR)	Not directly reported^a^	Preliminary outcome analysis
Schroder	2021	1,681	1,681	0%	No prior or current obstructive CAD	<2.25	4.5 years	CV death, MI, HF, stroke, revascularization	96	Dipyridamole	1.94 (HR)	Only women	Selected angina population

*Larger prognostic studies (n > 200) investigating coronary flow velocity reserve (CFVR) in angina patients*.

a *Sex-specific effect size was not reported as sex was not a significant predictor of outcome in multivariate-adjusted analysis*.

b *Eight hundred and nine patients (out of the total 5,577) had a new RWMA, indicating current obstructive CAD. The distribution of these 809 cases between the patient groups with or without prior obstructive CAD was not reported*.

*CAD, coronary artery disease; Cx, circumflex coronary artery; CVD, cardiovascular disease; CFVR, coronary flow velocity reserve; HF, heart failure; n/a, not available; MI, myocardial infarction; RCA, right coronary artery; RWMA, regional wall motion abnormality; TTDE, transthoracic Doppler echocardiography, UAP, unstable angina pectoris*.

A prognostic study from 2012 evaluated 272 patients with TTDE CFVR of the LAD and ICA for a composite endpoint of CVD death, MI, unstable angina, and HF (97). However, 38% had significant coronary stenosis of either the Cx or RCA coronary arteries. Low CFVR was significantly associated with the composite endpoint [adjusted odds ratio (OR) 5.6, *p* < 0.001]. Subgroup-specific ORs according to sex or presence of obstructive CAD were not reported, plausibly because neither obstructive CAD nor sex were individually related to the composite outcome. Another study from 2014 in the context of SE wall motion analysis also included both patients with suspected and previous obstructive CAD but excluded patients if they had any current regional wall motion abnormality (RWMA) (87). The composite outcome of CV death, MI, and revascularization were associated with low CFVR [adjusted hazard ratio (HR) 4.2, 95% CI 2.4–7.4] and active smoking, with a further increase in HR with lower CFVR cut-off <1.75 and <1.5. No HRs according to presence/absence of previous obstructive CAD or sex were reported, since these variables were not significantly related to the composite outcome in adjusted analyses.

The study of Cortigiani et al. has authored several studies evaluating angina patients using a SE protocol involving both wall motion score index analysis and CFVR measurement (33, 68, 98, 99). A large, pooled study included 5,577 angina patients with suspected or known obstructive CAD studied in a study period from 2003 to 2017 with a median follow-up of 1.7 years (68). The indication for the SE has suspected CAD in 63% and risk stratification of known CAD in 37%, wherein 59% of patients were men and the outcome was a composite of all-cause death and MI. There was a significant relationship between the composite outcome and low CFVR ≤ 2 (adjusted HR 3.26, *p* < 0.001) and RWMA on SE (adjusted HR 5.83, *p* < 0.002), while sex was not a significant predictor. The optimal prognostic CFVR cut-off value was comparable for men and women at 2.03 and 2.02, respectively, and was also comparable for patients with known CAD and suspected CAD at 2 and 2.03, respectively, as determined by receiver operating characteristics analyses. These results imply that low CFVR is likely a sex-independent significant predictor adding to the predictive accuracy offered by standard SE wall motion analysis, both in patients with and without known previous CAD.

Another recent study from 2019 is a prospective kind which included a cohort of angina patients and reported preliminary ad-interim composite outcome analyses on 1,867 patients with suspected or known CAD (88). RWMA and low CFVR ≤ 2 were significantly related to the composite outcome (adjusted HR 3.88, *p* < 0.001 and 1.6, *p* = 0.009, respectively). Patients were all-comers for SE and thus represented a wide clinical spectrum of patients from severe to no prior CV disease, and stress modalities including both dobutamine and exercise were used in addition to vasodilators.

In contrast to the more heterogeneous patient cohorts described above, a study published in 2021 prospectively enrolled only women with stable or unstable angina and no prior CAD or other cardiac diseases, and an invasive angiogram with no obstructive CAD (67). A cutpoint of < 50% stenosis was used to exclude significant stenosis, while fractional flow reserve measurements were regrettably not available. CFVR was obtained in 1,681 patients who were then followed for a median of 4.5 years for a composite outcome of CV death, MI, HF, stroke, or revascularization. Low CFVR < 2.25 was associated with increased risk of the composite outcome (HR 1.94, *p* = 0.001) and with all-cause mortality in secondary exploratory analysis. In subgroup analyses, the presence of a low CFVR remained significantly related to the composite outcome in both patients with and without non-obstructive atherosclerosis on the invasive angiogram (interaction *p* = 0.91), but there was significant interaction with BMI. CFVR was not associated with the composite outcome in patients with BMI > 30, possibly due to stress CFV acquisition difficulties and underestimation of CFVR in obese subjects. In short, low CFVR was also a significant prognostic marker in this highly selected patient cohort of women with relatively low event rates.

A meta-analysis conducted in 2018 about TTDE CFVR also included some of the studies referenced above and found that low CFVR was significantly associated with adverse outcomes in all four studies evaluating angina patients with no obstructive CAD (pooled relative risk 4.63 in patients with low CFVR, 95% CI 2.83–7.56) (66). There was no significant heterogeneity between studies, and the finding was robust in sensitivity analyses.

Regarding the optimal prognostic CFVR cut-off value, the continuous nature of the inverse association between CFVR and the adverse outcome is generally acknowledged. Several studies had shown that subdivision of patients into >2 subgroups according to CFVR value yields a dose-response relationship (67, 86, 87, 100, 101). Nonetheless, with the purpose of simplifying interpretation and perhaps to ease the transition of CFVR measurement from research to the clinical setting, most studies have reported their main findings according to a specific CFVR cut-off, most often 2 (68, 87, 88). The optimal cut-off value has been based on either receiver operating characteristics or related methods, then choosing the cut-off with the highest specificity and sensitivity (67, 68, 97). Complicating this discussion, however, is the fact that the optimal CFVR cut-off may be different in patients with or without prior obstructive CAD, patients with or without current wall motion abnormality, or angiographic stenosis, between men and women and between young and old patients. Although the large 2019 study by Cortigiani et al. involving more than 5,000 patients found that the cut-off 2 was optimal in most subgroups except the oldest old, this study also included patients with obstructive stenosis and short-term revascularization (68). Other studies have found optimal cut-off 2.25 or 2.4 (67, 97). In patients with no prior CAD and no current coronary stenosis, the CFVR is expectedly slightly higher than in patients with current stenosis, and the optimal CFVR cut-point identifying at-risk patients may therefore also be slightly higher, which could explain part of the cut-off discrepancy found in two of the largest CFVR studies (67, 68).

Moreover, the optimal cut-off is not necessarily the one that maximizes the sensitivity + specificity sum. A decrease in cut-point will necessarily involve a trade-off between the benefit of fewer false positives on the one hand and the disadvantage of more false negatives on the other, and the opposite will be the case if the cut-off point is elevated. Inherently, the issue of optimal cut-point also depends on weighing the benefits and risks of misclassifications and cannot be decided based on receiver operating characteristic statistics alone (101).

In summary, when reviewing the larger prognostic studies utilizing TTDE CFVR, only a few of them directly reported risk estimates according to sex or presence of known or current obstructive CAD. However, most of these studies found that sex and obstructive CAD status did not significantly modify the relation between low CFVR and adverse outcome, suggesting that low CFVR value is a significant and important marker of adverse prognosis in patients with no obstructive CAD regardless of sex.

## Other Modalities

The conception of the relationship between the degree of stenosis of the coronary artery lumen and resting and hyperemic flow, i.e., coronary flow reserve, was initially established using invasive Doppler quantification of coronary flow (9, 13, 14). Accordingly, initial attempts at ultrasound-based quantification of coronary flow, first using transesophageal echocardiographic evaluation of proximal coronary artery segments and later with TTDE of the mid-distal coronary artery segments, were evaluated against the gold standard of invasive CFR measurements (10, 11, 13). Several studies comparing TTDE CFVR and invasive Doppler CFR have generally shown good agreement, particularly when evaluating presence and degree of stenosis in patients with obstructive CAD (9, 11, 15, 39). Contemporary comprehensive invasive evaluation of coronary microvascular function include provocation with both adenosines assessing non-endothelium dependent microvascular dysfunction and acetylcholine assessing endothelium-dependent microvascular dysfunction and, importantly, macro- or microvascular vasospasm (102, 103). Several studies had estimated the prevalence and overlap of vasospasm and reduced microvascular dilatation. One study in 1,379 patients with stable angina and no obstructive CAD found acetylcholine infusion resulted in epicardial spasm in 26% and microvascular spasm in 33%. However, there were no flow measurements nor the assessment of vasodilatory capacity (CFR) in this study, hence, it remained unknown how many of the studied patients had a low CFR (104). Another study in 391 patients with angina and no obstructive CAD utilized both adenosine and acetylcholine and found 52% had isolated CMD (low CFR), 17% had isolated vasospasm, and 21% had a mixed endotype (102). In contrast, a recent study in 111 ANOCA patients found 63% had isolated vasospasm, only 3% had isolated impaired vasodilation, and 34% had a mixed endotype (105). In sum, a considerable subset of ANOCA patients should be expected to suffer from isolated vasospasm which will inherently go undetected by TTDE CFVR evaluation.

Positron emission tomography quantification of mutual and balanced force reduction (MBFR) has long been considered the non-invasive reference standard for CMD diagnosis (16, 25, 106). Since PET assesses global perfusion, while TTDE CFVR assesses flow velocity only in part of the coronary circulation, a comparison between positron emission tomography (PET) perfusion and CFVR measurements is of interest. This is partly because PET MBFR may have more limited availability and higher associated costs than TTDE. An early study in 10 healthy volunteers comparing CFVR and MBFR found an excellent correlation between the two, while a study in 86 obese patients with prior CAD found acceptable agreement between CFVR and MBFR, especially in patients without prior MI in the LAD territory (43, 47). In contrast, a more recent study in women with no obstructive CAD found a more modest agreement between the methods with MBFR results consistently higher than CFVR results (49). Internal reproducibility of the CFVR results were good and therefore not the cause of the modest agreement between methods, while PET examinations were not repeated. Interestingly, other studies of MBFR measurement found only modest agreement between repeated PET examinations (107, 108). Overall, it was concluded that CFVR and MBFR in ANOCA patients are not interchangeable, possibly due to the methodological differences.

Application of cMRI perfusion quantification at rest and during pharmacologic stress for the diagnosis of CMD is a research field in development with advantages similar to PET MBFR, e.g., evaluation of all coronary territories and high-quality assessment in obese individuals (16, 82, 109). The initial semi-quantitative CFR-surrogate myocardial perfusion reserve index (MPRI) is being further supplemented with fully quantitative perfusion measurements which may promote cardiac magnetic resonance as a beneficial future modality for comprehensive cardiac evaluation. Early studies in patients with ANOCA suggest subendocardial hypoperfusion during vasodilator stress (110), but only a few studies have compared cardiac magnetic resonance imaging and TTDE CFVR. One study measured both TTDE CFVR, PET MBFR, and cMRI-derived measures of cardiac fibrosis in women with ANOCA, and found no associations between fibrosis markers and presence of CMD (111). Directly comparing CMD diagnostic tests, a small study in 18 patients with ANOCA compared TTDE CFVR and cMRI perfusion and found a significant correlation between low CFVR measured in the LAD and stress perfusion defect in the LAD-territory on perfusion cMRI (112). Evidently, more studies are needed to determine whether CMD as detected by cMRI is correlated with low TTDE CFVR.

Cardiac CT perfusion imaging is a further emerging modality in the assessment of both the functional significance of epicardial stenosis and microvascular function, and several studies demonstrated a decrease in perfusion during pharmacologic stress in patients both with and without obstructive CAD (82, 113–115). Comparisons between CT perfusion and other modalities assessing CMD had primarily been made against PET MBFR (114, 116), and to our knowledge no studies had compared TTDE CFVR and cardiac CT perfusion results.

In summary, TTDE CFVR has shown a good correlation with invasive CFR measurements and ambiguous correlation with PET MBFR, while studies comparing TTDE with cMRI and cardiac CT perfusion were lacking.

## Therapeutic Interventions

Given that low CFVR at TTDE is an important predictor of adverse prognosis in angina patients there have been surprisingly few larger interventional studies in this patient group targeting CMD (25, 117). Consistently, management guidelines in ANOCA patients are unclear owing to lack of evidence-based data (118). A systematic review of treatment strategies in CMD covered most diagnostic modalities for evaluating CMD (invasive intracoronary Doppler, TTDE CFVR, and other non-invasive modalities), however, there were no restrictions about the studied cardiac condition (studies included patients with conditions such as ANOCA, obstructive CAD, hypertension, diabetes, and cardiomyopathy) (119). The authors found study methodology was too heterogeneous to allow meta-analysis, the sample size was generally small and the interventions were seldom placebo-controlled. Considering only a few studies utilizing TTDE CFVR in patients with ANOCA, there was a tendency toward an increase in CFVR from baseline after treatment with a statin, calcium-channel blocker, and ranolazine indicating it may be possible to improve microvascular function using medical drug interventions (120–123). Even so, overall the review found that no specific treatment was sufficiently well-documented to be recommended, and it was concluded that further stratified studies in larger patient cohorts are needed (119).

Three placebo-controlled interventional studies from our group investigated the effect of liraglutide (124), enalapril (125), and a multidisciplinary intervention including both drug therapy, low energy diet, and exercise (126), respectively, in ANOCA patients with low CFVR. None of these 3 studies found a significant increase in CFVR value at study completion, possibly in part due to small patient samples (*n* < 100 in all), however, there was not even an insignificant tendency toward an increase in CFVR value in the intervention groups.

The recent CorMica trial randomized patients with ANOCA to either standard care or a comprehensive interventional diagnostic procedure evaluating both coronary flow reserve and acetylcholine vasoreactivity. Patients were subsequently stratified to specific medical treatment based on the findings, and the study found an improvement in angina severity and quality of life in the intervention group (102). To our knowledge there have been no equivalent randomized trials to date utilizing CFVR for patient stratification. The ongoing large Warrior trial is investigating the benefit of a combined intervention consisting of both medication and lifestyle changes in ANOCA patients but without any stratification based on CMD presence or absence and will thus not resolve the question of targeting CMD (127).

In summary, larger intervention studies providing a stronger evidence base for treatment recommendations in ANOCA patients are needed, ideally investigating the effect on both improvement in microvascular function, e.g., CFVR, symptom amelioration, and prognostic benefit (25, 117, 119).

## Strengths and Limitations

The main strengths of TTDE CFVR in the evaluation of possible CMD include its non-invasive and inexpensive nature, whereas it is readily available at the bedside with no radiation exposure. Since the echocardiographic examination is already a mainstay in cardiologic evaluation, TTDE CFVR could be readily introduced in clinical practice. Importantly, low TTDE CFVR has been consistently associated with an adverse prognosis in diverse angina patient populations and specifically in the subgroup with no obstructive CAD. Several studies have reported feasibility >90% in the experienced hands, and this number may be even higher with the use of intravenous contrast in challenging cases. Inter- and intra-observer reproducibility has been high (8, 11, 12, 30).

The most important limitation of TTDE CFVR is the need for extensive training even in the hands of otherwise experienced echocardiographers. Examination quality continues to improve even after the performance of more than a 100 examinations, underlining the importance of sustained diligence, supervision, and adjustment to obtain high-quality reliable results (30). TTDE CFVR examination quality may be lower in obese patients (30). A large prognostic study in women indicated CFVR was not as strong a predictor of adverse prognosis in obese patients, possibly because maximum stress flow may be technically more difficult to obtain in obese subjects (67). Even so, TTDE CFVR is feasible in 90–95% of obese patients with acceptable examination quality (8, 30, 43, 128).

Another limitation is that only the LAD is accessible for evaluation in all patients, and consequently the obtained CFVR value is strictly the only representative of the LAD region. Even when it is ensured that any present LAD stenosis is non-significant (<50%) and FFR-negative (>0.8), it may be suspected maximal flow is still slightly attenuated, leading to possible underestimation of universal cardiac perfusion during stress. Furthermore, possible vasospasm and/or endothelium-dependent blunted vasodilation may only be assessed invasively using acetylcholine and is therefore not evaluated with TTDE. Comparisons with PET MBFR which is considered the present non-invasive reference standard have been ambiguous, and the use of TTDE CFVR may be challenged in future years by cMRI, PET MBFR, or cardiac CT perfusion in case these modalities become more accessible and are further developed in the decades to come.

## Conclusion and Future Perspectives

**Figure 2:**
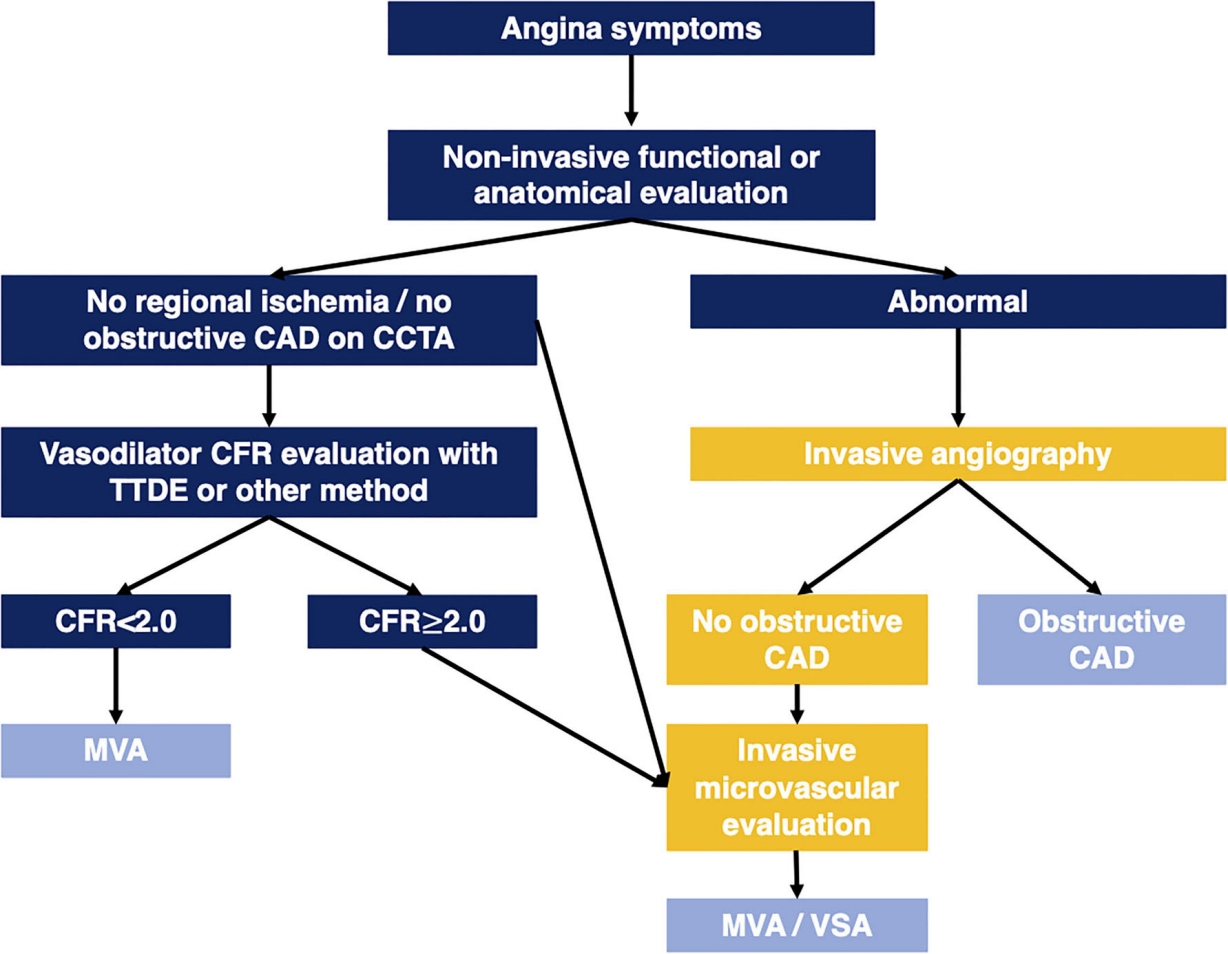
Algorithm illustrating diagnostic work-up in angina patients integrating CMD evaluation. CAD, coronary artery disease; CCTA, coronary computed tomography angiography; CFR, coronary flow reserve; MVA, microvascular angina; TTDE, transthoracic Doppler echocardiography; VSA, vasospastic angina.

Transthoracic Doppler echocardiography CFVR is feasible in the large majority, inexpensive, accessible, and carries prognostic information in ANOCA patients of both sexes. A low CFVR identifies angina patients who are at significantly higher risk of adverse outcomes. Given the large number of angina patients with negative non-invasive diagnostic work-up or absence of significant epicardial stenosis at ICA, there is a need for diagnostic tools that are capable of large-scale screening of patients. Although PET MBFR and stress cMRI are becoming increasingly available in many countries the current capacity only allows evaluation of a small part of the large population of ANOCA patients. A suggested algorithm for diagnostic work-up of angina patients integrating CMD evaluation is shown in Figure 2.

**Figure 3:**
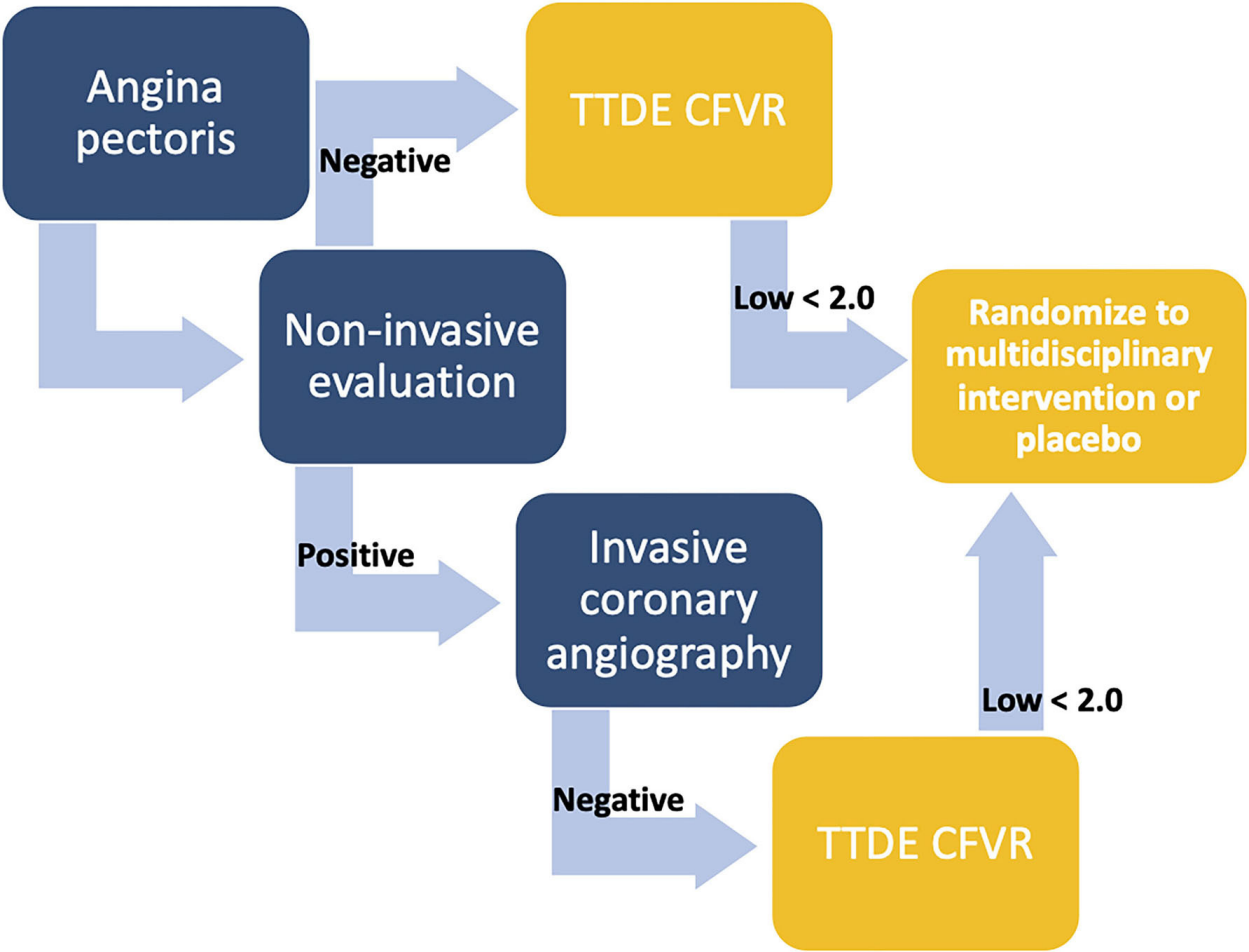
Algorithm illustrating proposed patient cohorts for inclusion in future interventional studies. Coronary flow velocity reserve (CFVR) evaluation in angina patients is suggested either after a negative non-invasive test (functional or anatomical) or following a positive non-invasive test and an invasive coronary angiography with no significant stenosis. In case of a low CFVR, e.g., <2, participation in a randomized placebo-controlled intervention study is suggested. CFVR, coronary flow velocity reserve; TTDE, transthoracic Doppler echocardiography.

Ultimately, implementation of new diagnostic tools should be based on their potential to change treatment recommendations and patient outcomes, and current evidence on beneficial treatment interventions in patients with CMD is lacking. This is reflected in the latest guidelines on chronic coronary syndromes which only briefly address microvascular disease. Prior to the potential implementation of CFVR evaluation in guidelines and clinical practice, randomized interventional trials stratifying patients according to low or normal CFVR must clarify whether it is possible to improve patient outcomes, ameliorate symptoms and improve microvascular function in ANOCA patients. We proposed future large-scale studies recruiting ANOCA patients with either a negative non-invasive diagnostic test or an ICA with no significant obstruction and a low CFVR, randomizing them to pharmacological therapy and lifestyle interventions, with a primary outcome of symptom severity and prognosis, and secondary outcome of increased CFVR on follow-up (Figure 3). An equivalent study using invasive, comprehensive vasofunction assessment has shown that it was possible to reduce symptom severity (102). However, it should be clarified whether this is also the case with TTDE CFVR-guided therapy.

Regarding the choice of modality for CMD assessment, it may be argued implementation of TTDE CFVR is more straightforward in countries and hospitals with a tradition for SE and wall motion analysis in the evaluation of patients with suspected CAD. Even so, with proper education and training, it is feasible for experienced echocardiographers to include TTDE CFVR in their diagnostic armamentarium. Invasive evaluation of microvascular function, the only modality with the capacity for identification of vasospasm, will most likely remain an option only in a smaller, selected subgroup of angina patients. Both TTDE CFVR, PET MBFR, stress cMRI, or perhaps cardiac CT perfusion may appear as the non-invasive diagnostic test of choice for microvascular assessment in the years to come, likely based on local expertise and availability, and future studies evaluating the benefit of CMD assessment.

## Author Contributions

JS and EP were responsible for the drafting and final revision of the manuscript. Both authors contributed to the article and approved the submitted version.

## Conflict of Interest

The authors declare that the research was conducted in the absence of any commercial or financial relationships that could be construed as a potential conflict of interest.

## Publisher's Note

All claims expressed in this article are solely those of the authors and do not necessarily represent those of their affiliated organizations, or those of the publisher, the editors and the reviewers. Any product that may be evaluated in this article, or claim that may be made by its manufacturer, is not guaranteed or endorsed by the publisher.

## Supplementary Material

Supplementary Video 1 Transthoracic Doppler echocardiography, mid-distal LAD at rest. Transthoracic Doppler echocardiogram loop in a modified 2/3-chamber view demonstrating a low-velocity color Doppler signal in the mid-distal left anterior descending artery at rest.

Supplementary Video 2 Transthoracic Doppler Echocardiography, mid-distal LAD during vasodilator stress. Transthoracic Doppler echocardiogram loop in a modified 2/3-chamber view demonstrating the mid-distal left anterior descending artery (LAD) during adenosine vasodilator stress. A smaller coronary branch is visible near the LAD (red signal), while the LAD displays an aliasing high-velocity color Doppler signal above the set velocity range of 0.17 to −0.17.

## Abbreviations

ANOCA: angina with no obstructive coronary artery disease
CAD: coronary artery disease
CFR: coronary flow reserve
CFVR: coronary flow velocity reserve
CMD: coronary microvascular dysfunction
cMRI: cardiac magnetic resonance imaging
Cx: circumflex coronary artery
ICA: invasive coronary angiography
PET: positron emission tomography
RCA: right coronary artery
RWMA: regional wall motion abnormality
SE: stress echocardiography
TTDE: transthoracic Doppler echocardiography.
